# Implementation of an Interdisciplinary, Team-Based Complex Care Support Health Care Model at an Academic Medical Center: Impact on Health Care Utilization and Quality of Life

**DOI:** 10.1371/journal.pone.0148096

**Published:** 2016-02-12

**Authors:** Christine Ritchie, Robin Andersen, Jessica Eng, Sarah K. Garrigues, Gina Intinarelli, Helen Kao, Suzanne Kawahara, Kanan Patel, Lisa Sapiro, Anne Thibault, Erika Tunick, Deborah E. Barnes

**Affiliations:** 1 Department of Medicine, University of California San Francisco, San Francisco, California, United States of America; 2 Tideswell at UCSF, Division of Geriatrics, University of California San Francisco, San Francisco, California, United States of America; 3 UCSF Health, University of California San Francisco, San Francisco, California, United States of America; 4 Geriatrics, Palliative and Extended Care Service, San Francisco Veterans Affairs Medical Center, San Francisco, California, United States of America; 5 Research Service, San Francisco Veterans Affairs Medical Center, San Francisco, California, United States of America; University of Ottawa, CANADA

## Abstract

**Introduction:**

The Geriatric Resources for the Assessment and Care of Elders (GRACE) program has been shown to decrease acute care utilization and increase patient self-rated health in low-income seniors at community-based health centers.

**Aims:**

To describe adaptation of the GRACE model to include adults of all ages (named Care Support) and to evaluate the process and impact of Care Support implementation at an urban academic medical center.

**Setting:**

152 high-risk patients (≥5 ED visits or ≥2 hospitalizations in the past 12 months) enrolled from four medical clinics from 4/29/2013 to 5/31/2014.

**Program Description:**

Patients received a comprehensive in-home assessment by a nurse practitioner/social worker (NP/SW) team, who then met with a larger interdisciplinary team to develop an individualized care plan. In consultation with the primary care team, standardized care protocols were activated to address relevant key issues as needed.

**Program Evaluation:**

A process evaluation based on the Consolidated Framework for Implementation Research identified key adaptations of the original model, which included streamlining of standardized protocols, augmenting mental health interventions and performing some assessments in the clinic. A summative evaluation found a significant decline in the median number of ED visits (5.5 to 0, p = 0.015) and hospitalizations (5.5 to 0, p<0.001) 6 months before enrollment in Care Support compared to 6 months after enrollment. In addition, the percent of patients reporting better self-rated health increased from 31% at enrollment to 64% at 9 months (p = 0.002). Semi-structured interviews with Care Support team members identified patients with multiple, complex conditions; little community support; and mild anxiety as those who appeared to benefit the most from the program.

**Discussion:**

It was feasible to implement GRACE/Care Support at an academic medical center by making adaptations based on local needs. Care Support patients experienced significant reductions in acute care utilization and significant improvements in self-rated health.

## Introduction

‘Complex care’ refers to patients with health care needs that are complicated by significant medical and psychosocial factors, such as multiple chronic conditions and comorbid physical and mental health conditions.[1, 2] Patients with complex care needs are a major driver of health care costs, with 10% of patients accounting for 64% of total health care costs.[3, 4]

The Geriatric Resources for the Assessment and Care of Elders (GRACE) program is a health care delivery model that was developed to improve care while controlling costs for older patients with complex care needs.[5] GRACE was designed to serve as a support system between patients/caregivers and the primary care provider (PCP). The model includes a nurse practitioner/social worker (NP/SW) team that performs comprehensive, structured assessments in patients’ homes and then meets as part of a larger interdisciplinary team that includes a geriatrician, mental health liaison and pharmacist. The driver of GRACE Team Care is an individualized care plan developed by the GRACE team based on the initial in-home assessment and the patient’s goals of care. The care plan is built using the GRACE Protocols for common geriatric conditions. A few of the GRACE Protocols are Cognitive Impairment, Difficulty Walking/Falls, Health Maintenance, and Advance Care Planning. These care protocols and corresponding Team Suggestions for evaluation and management are a combination of medical and psychosocial interventions and based on published practice guidelines. The GRACE Protocols provide a checklist to ensure a standardized and state-of-the-art approach to care. The GRACE Team works alongside the patient’s primary care team to implement the care plan and modify it as needed over time. Additional information is available on the GRACE Team Care website: http://graceteamcare.indiana.edu/home.html.

In a randomized, controlled trial, patients at high risk for hospital admission who received GRACE team care versus a ‘usual care’ control group had decreased healthcare utilization and healthcare costs; improved quality of care; increased patient and provider satisfaction; and improved quality of life.[6, 7]

The primary objective of the current study was to evaluate the adaptation and implementation of GRACE at an urban academic medical center. Our evaluation was informed by the Consolidated Framework for Implementation Research (CFIR) and was performed as a partnership among clinical and research teams.[8] We performed a process evaluation to assess fidelity to the original program, adaptations for the current environment, and barriers and facilitators encountered. As described in detail below, one of the key adaptations was to include adult patients 18 years and older who met enrollment criteria; therefore, the program was renamed Care Support to reflect this more inclusive age range. We also performed a summative evaluation to examine the impact of implementation on health care utilization and patient quality of life.

## Methods

### Setting and Patient Population

The setting for this implementation study was four primary care medical clinics at a large urban academic medical center. Within each clinic, ‘high-risk’ patients—defined as patients with ≥5 emergency department (ED) visits or ≥2 inpatient hospitalizations in the past 12 months—were identified based on lists of recent admissions and direct referrals. These high-utilizing patients were then vetted by PCPs for appropriateness of enrollment taking into consideration the patients’ needs, current resources and potential for engagement. For example, PCPs may have decided that Care Support was not necessary for patients whose high utilization was appropriate for their medical condition and were already well-supported, who were connected with another team providing aggressive care management, or who were rapidly declining and unlikely to benefit. Patients who were approved by their PCPs were placed on the Care Support Registry. To target enrollment to those patients with persistent high utilization, after their next ED visit or hospitalization, PCPs were asked to contact patients directly to assess their interest in participating in Care Support. Patients who agreed were then contacted by the Care Support team to schedule an initial in-home assessment by the NP/SW team.

This study was approved by the UCSF Institutional Review Board (IRB). Because our study involved retrospective review of data that had been previously collected as part of clinical care and quality improvement, patient informed consent and HIPAA authorization were waived. The UCSF IRB approved release of a limited dataset for this publication, which is included as supplementary information (S1 Appendix). To minimize the risk of loss of privacy for older patients, age has been truncated to a maximum value of 89 years. We do not have IRB approval to provide specific ages for patients age 90 years or older.

### Overview of Care Support

At the initial in-home assessment, the NP/SW team performed a comprehensive evaluation of the patient’s needs and available resources. In some cases, the initial assessment was performed in the clinic or by phone if the patient declined the in-home assessment. The initial assessment was then discussed with the larger interdisciplinary team that included a geriatrician, mental health liaison and pharmacist. An individualized care plan was created for each patient that included activation of specific care protocols, which were then reviewed and modified as needed by the primary care physician. Follow-up visits were typically conducted by phone unless a follow-up home visit was deemed critical to the patient’s ongoing well-being. Patients continued to receive regular telephone contacts as needed and were discussed at interdisciplinary team meetings on a quarterly basis or more frequently if needed. After hours support was provided by the primary care team.

### Process Evaluation

#### Patients, Process and Fidelity

A dedicated Care Support database included information gathered by the NP/SW team at the initial home assessment, such as patient demographic information, insurance status, falls, housing status, depressive symptoms (PHQ-4),[9] and dependency in basic and instrumental activities of daily living (ADL/IADL).[10, 11] Data also were collected on process measures including time between key milestone events (e.g., patient enrollment and initial home assessment); number and type of protocols recommended; and number of face-to-face and telephone patient contacts over time. Additional data on diagnoses and use of alcohol and tobacco were assessed by the clinical team from the electronic medical record. Descriptive statistics were used to summarize the patient population, implementation process and fidelity to the original GRACE model. In addition, age groups (<65 vs. ≥65 years) were compared using t-tests, Wilcoxon rank-sum tests or Chi-square tests as appropriate.

#### Adaptations, Barriers and Facilitators

Semi-structured group and individual interviews were performed with Care Support team members in July 2014, approximately 14 months after the first patient had been enrolled. Interview questions were based on the CFIR conceptual model and focused on identifying adaptations to the original GRACE model and barriers and facilitators to implementation. In addition, team members were asked to reflect on the characteristics of patients that appeared to benefit the most and the least from participation in the program. One investigator who was not part of the clinical team (DB) took detailed notes and performed a thematic analysis which was then reviewed and confirmed by clinical team members.

### Summative Evaluation

#### Health care Utilization

Utilization data including dates of ED visits and hospital admissions for Care Support patients both 6 months prior to enrollment and 6 months after enrollment were extracted from the electronic medical record. Length of stay during hospital admissions was also determined. Given differential dates of enrollment and lengths of follow-up, we calculated ED and hospitalization rates per 1,000 observation days. Because the distributions were highly skewed, we compared pre- and post-enrollment rates using non-parametric Wilcoxon signed-rank tests. In addition, we compared the proportions of patients with zero ED visits or hospitalizations pre- and post-enrollment using McNemar’s test. Analyses of health care utilization were restricted to patients with >25 days of follow-up to ensure that the median number of observation days was similar during the pre- and post-enrollment observation periods. Patients also were asked about ED visits and hospital admissions outside UCSF at the initial home assessment and every 3 months thereafter (see below).

#### Patient Self-Rated Health

The NP/SW team assessed patient self-rated health at the initial home visit and either in person or by telephone every 3 months thereafter based on current health status (poor, fair, good, very good, excellent) and health status compared to three months ago (much worse, somewhat worse, about the same, somewhat better, much better). ‘Good’ current self-rated health at each time point was defined as reporting good, very good or excellent health. ‘Better’ health at each time point was defined as reporting somewhat or much better health over the past 3 months. Changes compared to baseline values were determined using McNemar’s test.

## Results

**Fig 1:**
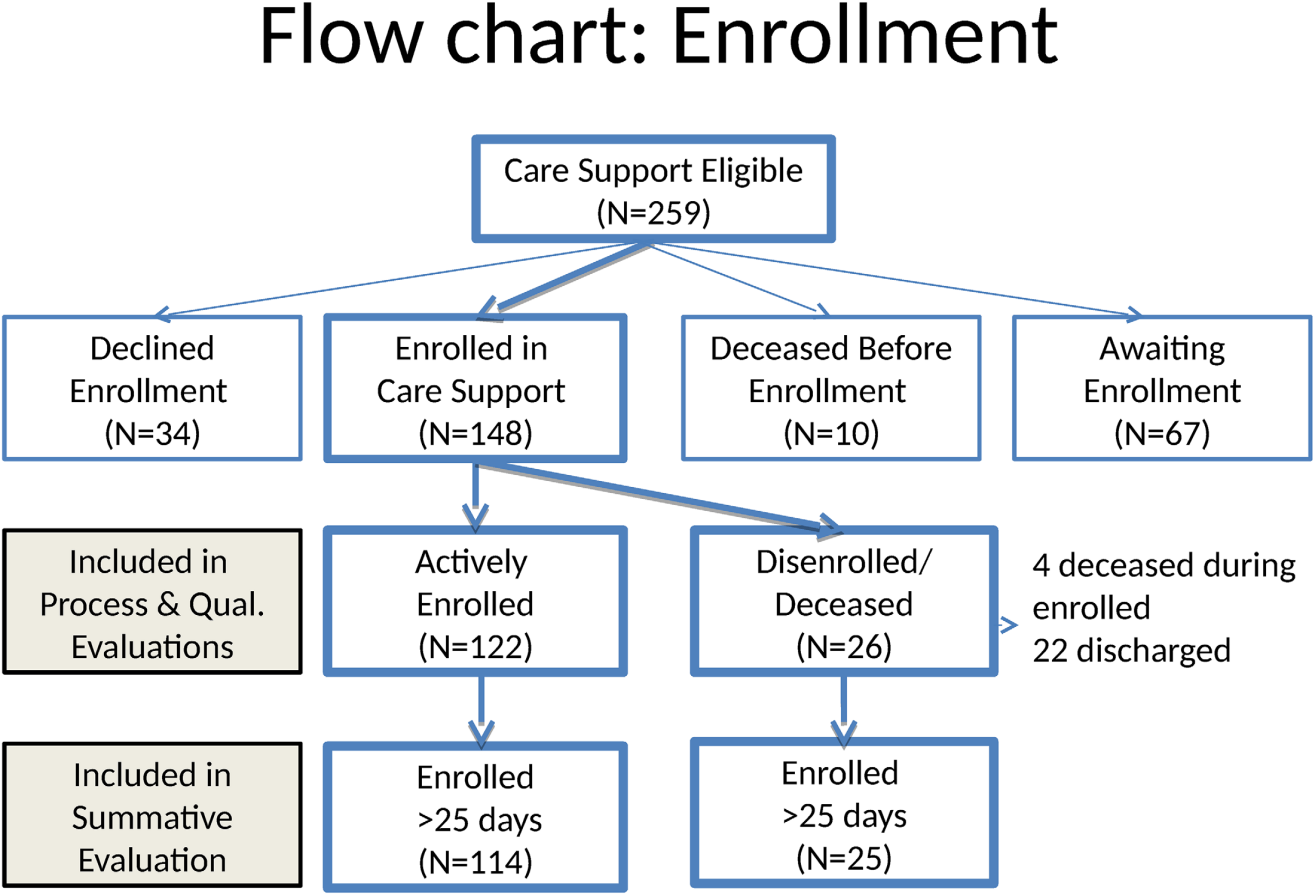
Flow of Patients.

The flow of patients is shown in Fig 1. A total of 259 patients were identified as being eligible for Care Support. Of these, 148 were enrolled from 4/29/2013 to 5/31/2014 while 34 declined enrollment, ten died before enrollment, and 67 were placed on a waitlist. There were a variety of reasons for disenrollment, including: 1) patient request, 2) team assessment that patient goals for the program had been met and 3) inability to engage patient due to lack of interest or to reach patient by phone or in person. The process evaluation included all 148 patients who enrolled in Care Support, of whom 26 disenrolled (22 were discharged, 4 died) during the observation period. The summative evaluation was restricted to the 139 patients with >25 days of follow-up, of whom 25 had disenrolled or died during the observation period.

### Process Evaluation

#### Patients, Process and Fidelity

Demographic characteristics of Care Support patients are shown in Table 1. Patients had a mean age of 65 years at enrollment; 60% were women, 85% had at least a high school degree, and 26% were living alone. Younger patients were more likely than older patients to report fair/poor self-rated health (85% vs. 55%, p<0.001) and had more depressive symptoms (median: 4 vs. 1, p<0.001) but were less likely to be dependent in one or more IADLs (37% vs. 72%, p<0.001) or ADLs (19% vs. 6%, p = 0.02).

**Table 1:** Characteristics of Care Support Patients.

Characteristics	Value /Sub-Group	Overall (n = 148)	Age < 65 (n = 67)	Age ≥ 65 (n = 81)	p-value
Age at enrollment	Mean ± SD	65.5 ± 18.5	49.1 ± 12.7	79.0 ± 9.3	<0.001
Female sex	Number (%)	89 (60.1)	35 (52.2)	54 (66.7)	0.074
Education	Number (%)				
	<High School	22 (15.5)	7 (10.9)	15 (19.2)	0.301
	High School	32 (22.5)	17 (26.6)	15 (19.2)	
	>High School	88 (62.0)	40 (62.5)	48 (61.5)	
Living alone	Number (%)	37 (25.7)	13 (20.0)	24 (30.4)	0.050
Insurance status	Number (%)				
	Medicare A/B	90 (60.8)	19 (28.4)	71 (87.7)	<0.001
	Medicaid	82 (55.4)	44 (65.7)	38 (46.9)	0.022
	Private	59 (39.9)	22 (32.8)	37 (45.7)	0.112
Self-rated health	Number (%)				
	Fair/Poor	96 (68.6)	55 (84.6)	41 (54.7)	<0.001
Medical diagnoses	Number (%)				
	Hypertension	70 (54.3)	29 (52.7)	41 (55.4)	0.763
	Diabetes	45 (34.9)	20 (36.4)	25 (33.8)	0.761
	Renal disease	42 (32.6)	13 (23.6)	29 (39.2)	0.062
	CHF	35 (27.1)	8 (14.5)	27 (36.5)	0.006
	COPD	34 (26.4)	20 (36.4)	14 (18.9)	0.026
Depressive symptoms	Median (range)	2 (0–12)	4 (0–12)	1 (0–11)	<0.001
Alcohol consumption	Number (%)				
	None	95 (70.4)	41 (66.1)	54 (74.0)	.234
	Moderate	23 (17.0)	11 (17.7)	12 (16.4)	
	High	17 (12.6)	10 (16.1)	7 (9.6)	
Smoking	Number (%)				
	Current	26 (19.7)	17 (28.8)	9 (12.3)	0.022
	Former	44 (33.3)	21 (35.6)	23 (31.5)	
	Never	62 (47.0)	21 (35.6)	41 (56.2)	
≥ 1 IADL dependency	Number (%)	81 (56.3)	24 (36.9)	57 (72.2)	<0.001
≥ 1 ADL dependency	Number (%)	19 (13.2)	4 (6.2)	15 (19.0)	0.024
Fall, past 6 months		57 (41.6)	22 (35.5)	35 (46.7)	0.186

Abbreviations: ADL, activities of daily living; CHF, congestive heart failure; COPD, chronic obstructive pulmonary disease; IADL, instrumental activities of daily living; SD, standard deviation. Percentages calculated excluding missing data. Data missing as follows: education (n = 6), living alone (n = 4), self-rated health (n = 8), medical diagnosis (n = 19), PHQ4 (n = 21), alcohol (n = 13), smoking (n = 16), IADL (n = 4), ADL (n = 4), falls (n = 11).

Process measures and fidelity to the original GRACE model are shown in Table 2. In general, Care Support adhered to the process goals of GRACE with high fidelity. An average of six standardized protocols were activated, the most common of which were chronic condition self-management (95%), social service coordination (87%) and advanced care planning (83%). Mental health protocols were activated more often in younger than older patients (70% vs. 48%, p = 0.007). The Care Support team interacted with patients an average of once in person and three times by phone during the first 30 days of enrollment.

**Table 2:** Process Measures and Fidelity to GRACE Model.

GRACE Process Target Achieved	Value /Sub-Group	Overall (n = 148)	Age < 65 (n = 67)	Age ≥ 65 (n = 81)	p-value
In-home assessment ≤ 15 days after enrollment	Number (%)	146 (98.6)	66 (98.5)	80 (98.8)	--
Team conference ≤ 15 days after in-home assessment	Number (%)	144 (97.3)	66 (98.5)	78 (96.3)	0.196
Team conference included:	Number (%)				
	Geriatrician	144 (97.3)	64 (95.5)	80 (98.8)	0.117
	Mental health	134 (90.5)	60 (89.6)	74 (91.4)	0.728
	Pharmacist	123 (83.1)	55 (82.1)	68 (84.0)	0.783
Care plan reviewed with PCP ≤ 15 days	Number (%)	139 (93.9)	63 (94.0)	76 (93.8)	0.463
Care plan reviewed with patient ≤ 1 month	Number (%)	142 (95.9)	65 (97.0)	77 (95.1)	--
Patient contacted ≤ 5 days after ED visit or discharge *	Number (%)	38 (79.2)	30 (93.8)	8 (50.0)	<0.001
**Other Process Measures**					
Protocols activated	Mean ± SD	5.6 ± 1.7	5.4 ± 1.7	5.7 ± 1.6	0.283
Protocol types activated	Number (%)				
	Chronic condition	141 (95.3)	64 (95.5)	77 (95.1)	0.895
	Social service	131 (88.5)	61 (91.0)	70 (86.4)	0.380
	Advanced care planning	124 (83.8)	62 (92.5)	62 (76.5)	0.009
	Mental health	86 (58.1)	47 (70.1)	39 (48.1)	0.007
	Medication management	59 (39.9)	21 (31.3)	38 (46.9)	0.054
	Mobility/falls	58 (39.2)	14 (20.9)	44 (54.3)	<0.001
	Caregiver support	56 (37.8)	19 (28.4)	37 (45.7)	0.031
Face-to-face contacts, first 30 days	Mean ± SD	1.0 ± 1.1	1.0 ± 1.1	1.0 ± 1.1	0.837
Telephone contacts, first 30 days	Mean ± SD	2.6 ± 2.2	2.7 ± 1.8	2.6 ± 2.5	0.742

Abbreviations: ED, emergency department; PCP, primary care physician. Missing data included in denominator for percentages. Data missing as follows: team conference (n = 2), care plan reviewed (n = 6), patient contacted ≤ 5 days after ED visit (n = 6), face-to-face contacts (n = 6), telephone contact (n = 6).

*Restricted to patients with at least one ED visit or discharge during the first 30 days.

#### Adaptations, Barriers and Facilitators

Several adaptations were made to accommodate the internal environment (Table 3). Enrollment criteria were relaxed to include patients of all ages who PCPs felt could potentially benefit from the interventions; standardized protocols were adapted based on the needs of this expanded patient population; some assessments were performed in the clinic or by phone rather than at home based on patient preferences. Despite these adaptations, most core elements of the GRACE model were not changed, including the comprehensive NP/SW assessment, development of individualized care plans in consultation with an interdisciplinary team, review and approval by the PCP, and activation of standardized care protocols.

**Table 3:** Core Components of GRACE and Adaptations for Care Support.

Original GRACE Model	Adaptations for Care Support
Age ≥ 65	No age restriction
Comprehensive in-home assessment by NP/SW team	Some assessments performed in clinic
Individualized care plan developed with interdisciplinary team	--
Approval of care plan by primary care physician	--
Activation of standardized care protocols	Protocols simplified and streamlined

The key barriers to Care Support implementation included: changes in the enrollment criteria as the program evolved, which made it more difficult for PCPs to identify appropriate patients for enrollment and sometimes resulted in frustration with the program and the process; limited initial access to mental health services, which made it difficult to support patients with more severe mental illness; being spread out at multiple sites, which made meeting and communication more difficult; and the size of the patient panel, which sometimes limited the amount of time available to address the needs of each patient.

The key facilitators identified included: the GRACE protocols, which provided structured and adaptable templates and enabled the team to more efficiently manage patients with complex care needs; the home visit, which provided the team with key insights into the real-world issues and day-to-day needs of each patient in their own environment; the comprehensive assessment, which provided a complete picture of all of the patient’s needs and the interdisciplinary team, which enabled incorporation of a wide range of perspectives. In addition, being embedded in the primary care clinics enabled the Care Support team to build greater rapport with PCPs and to meet patients at their clinic visits, thereby increasing the frequency of in-person “touches” and increasing the ability to implement interventions quickly and strengthening relationships with patients.

Finally, several patient profiles were described as seeming to benefit the most from Care Support. 1) In patients with high, resource-intensive needs—particularly those with multiple complex conditions and poor care coordination—the Care Support team was able to hand-pick a team of expert care providers, augment self-management and caregiver support and assist with care coordination in order to provide these complex patients with optimal care. 2) In patients with little community support—particularly those who were living alone, non-trusting of the medical system or resistant to care—the Care Support team was able to build trust using a more personal approach and to help these patients develop their self-management skills once trust had been gained. 3) In patients with mild anxiety who were using the ED to address an array of symptom concerns, the Care Support team was able to develop personal relationships in which patients would call them before going to the ED, so that the Care Support team could provide reassurance when indicated and minimize unnecessary ED visits.

In contrast, patients who seemed to benefit the least from Care Support exhibited different patient profiles. In particular, the NP/SW teams felt that they did not have the training to provide optimal care for patients with more severe or very complex mental health needs, such as those with active substance abuse, alcoholism or personality disorders. It was felt that these patients would be better served by either referral to specialty mental health professionals or integration of mental health professionals into the team. In addition, some patients were not ready to engage with the Care Support team or learn self-management skills.

### Summative Evaluation

#### Health care Utilization

By design, all patients had 182 observation days during the 6-month period before enrollment in Care Support. After enrolling in Care Support, the number of observation days varied widely (median: 180; range: 26–397): patients were enrolled on an ongoing basis from 4/29/2013–5/31/2014; therefore, those enrolled earlier had more time available for follow-up. However, the median number of observation days in the pre- and post-Care Support periods did not differ significantly (p = 0.54).

**Fig 2:**
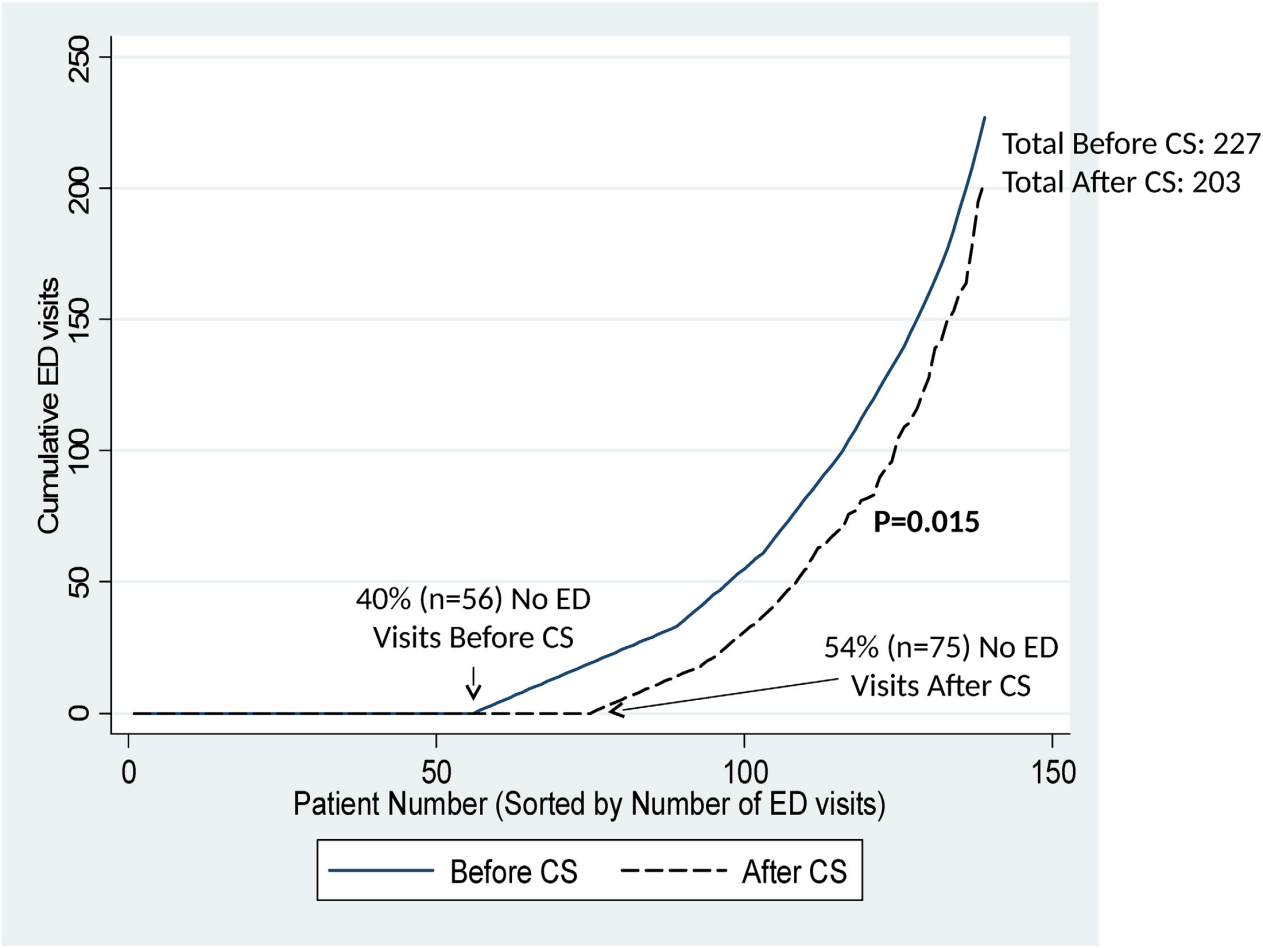
Cumulative Number of Emergency Department (ED) Visits in Care Support (CS) Patients Before and After Enrollment. The cumulative number of ED visits is shown as a function of patient number (sorted by number of ED visits) during the 6 months before enrollment in Care Support (solid line) and the 6 months after enrollment (dashed line). The proportion of patients with zero ED visits increased significantly from 40% pre-enrollment to 54% post-enrollment (McNemar’s test, p = 0.015).

The median number of ED visits/1000 observation days declined significantly from 5.5 (range: 0–54.9) before Care Support to 0 (range: 0–87.0) after Care Support enrollment (p = 0.015). As shown in Fig 2, this difference was primarily attributable to a significant increase in the proportion of patients with zero ED visits before and after enrollment (40% vs. 54%, p = 0.015). The total number of ED visits in these patients was 227 before Care Support and 203 after Care Support.

**Fig 3:**
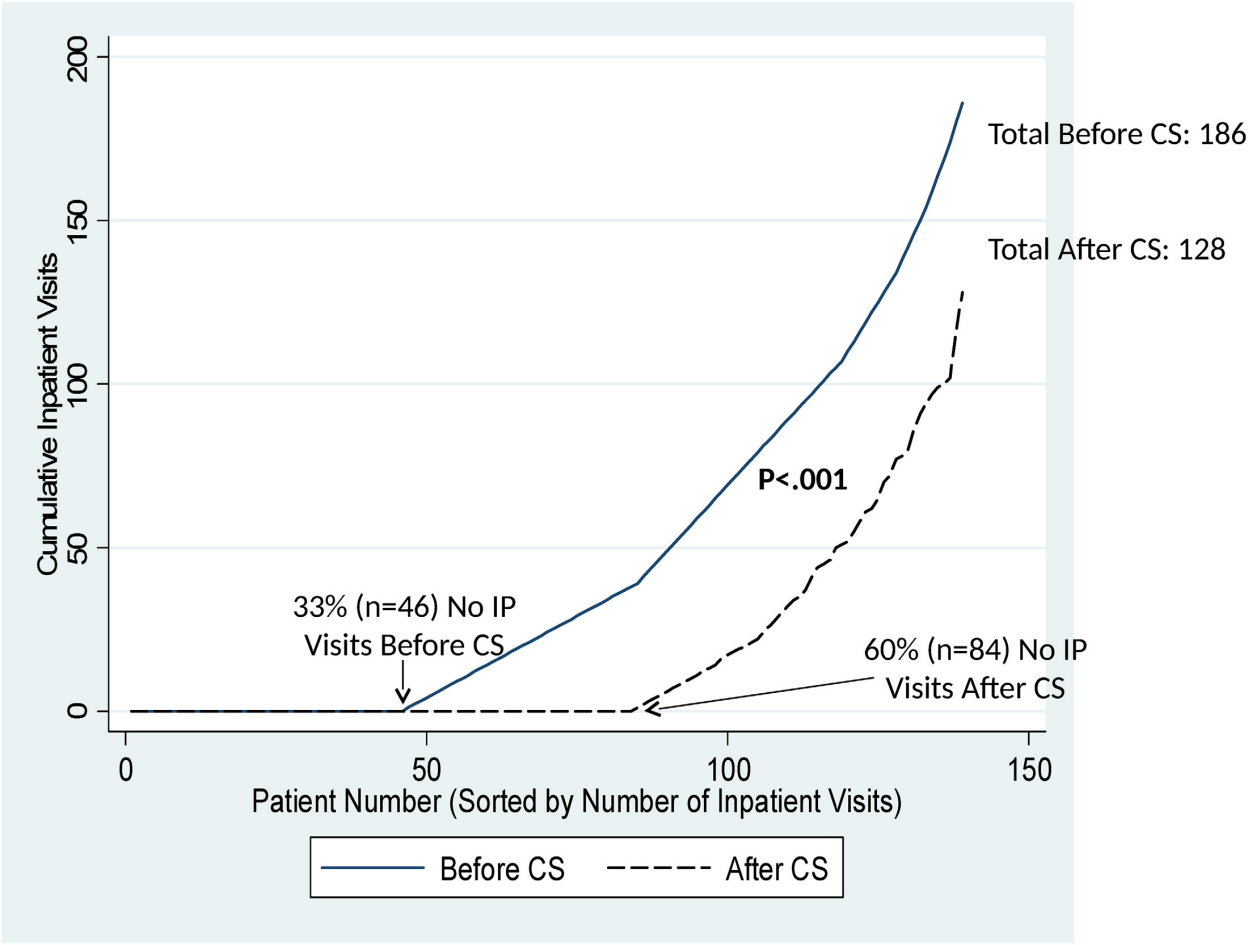
Cumulative Number of Inpatient Visits (IP) Visits in Care Support (CS) Patients Before and After Enrollment. The cumulative number of IP visits is shown as a function of patient number (sorted by number of IP visits) during the 6 months before enrollment in Care Support (solid line) and the 6 months after enrollment (dashed line). The proportion of patients with zero IP visits increased significantly from 33% pre-enrollment to 60% post-enrollment (McNemar’s test, p<0.001).

Similarly, the median number of hospitalizations/1000 observation days declined significantly from 5.5 (range: 0–33.0) to 0 (range: 0–43.0) before and after Care Support enrollment (p<0.001). This difference also was primarily attributable to the proportion of patients with zero hospitalizations, which nearly doubled from 33% before Care Support to 60% after Care Support (p<0.001, Fig 3). The total number of hospitalizations in these patients was 186 before Care Support and 128 after Care Support. In those who were hospitalized, median length of stay did not differ before (median: 6 days; range: 1–57) vs. after (median: 5; range: 1–72) Care Support (p = 0.25). There was no evidence of difference based on age.

There were relatively few non-UCSF ED visits and hospitalizations reported. Patients reported 20 non-UCSF ED visits during the 6 months before enrollment in Care Support compared to 16 after (p = 0.56). There was a significant decline in non-UCSF ED admissions, with 16 reported prior to enrollment in Care Support and 4 reported after enrollment (p = 0.008).

#### Patient Self-Rated Health

**Fig 4:**
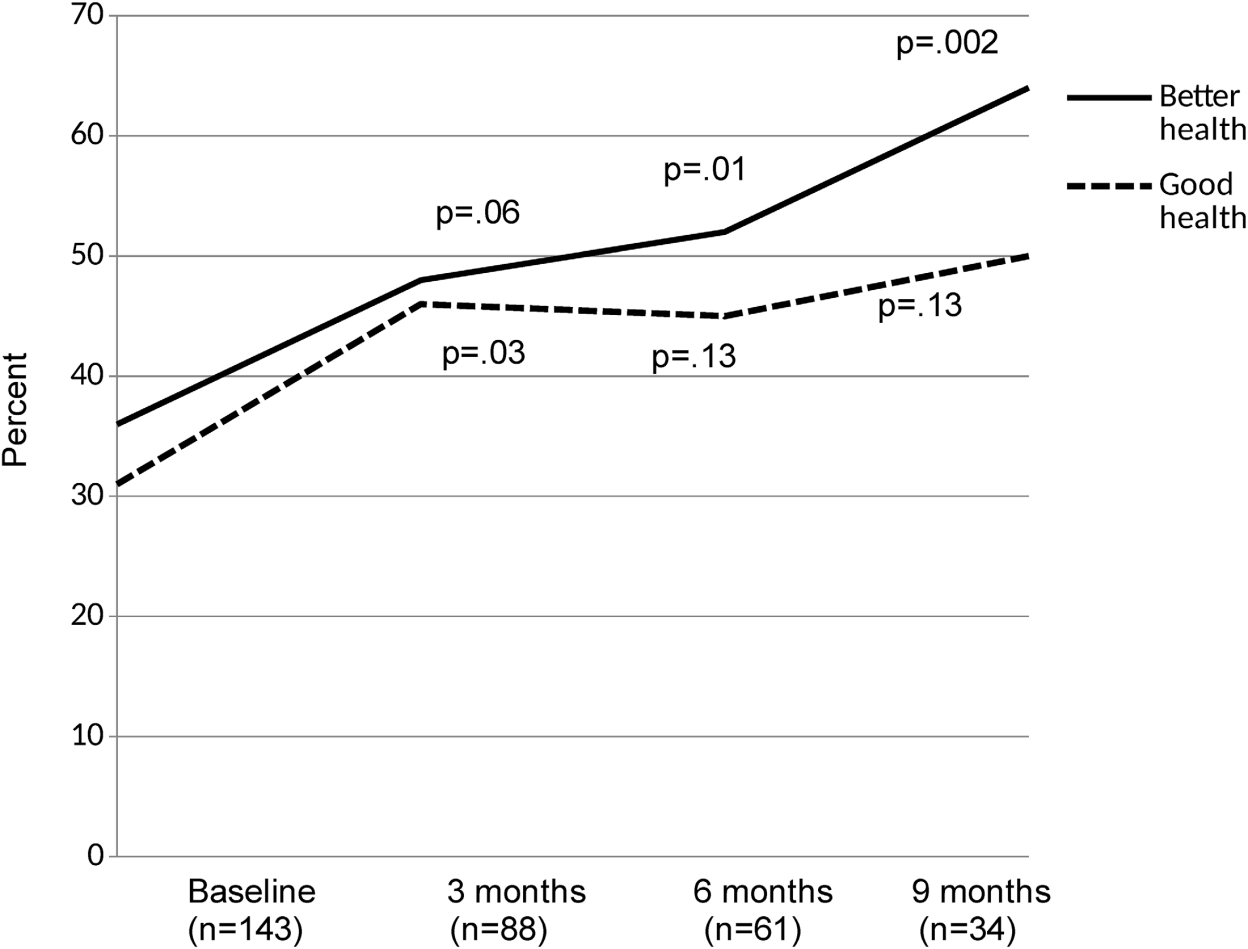
Changes in Self-Rated Health in Care Support Patients Over Time. The percentage of patients who self-reported good health (defined as good, very good or excellent versus fair or poor) is shown in red, while the percentage of patients who self-reported that their health was better than 3 months ago (defined as somewhat or much better versus about the same, somewhat worse or much worse) is shown in blue. P-values are based on McNemar’s test for paired proportions compared to baseline values.

The proportion of patients who rated their health as good, very good or excellent increased over time from 31% at the time of enrollment in Care Support to 50% after 9 months, although this was statistically significant only at 3 months (Fig 4). Similarly, the proportion of patients who reported that their health was somewhat or much better than three months ago increased from 36% at enrollment to 64% at 9 months (p = 0.002) (Fig 4).

## Discussion

In this study, we used the CFIR conceptual model to evaluate the process and impact of implementation of the evidence-based GRACE model at an urban, academic medical center. Importantly, one of the key adaptations to meet the needs of our medical center was to expand the program to include high-utilizing and high-need adult patients of all ages rather than restricting enrollment to older patients, which resulted in changing the name to Care Support, revising protocols and addressing an array of patient concerns beyond traditional geriatric syndromes. None-the-less, most core components of the program remained consistent with the original model, including comprehensive in-home assessments by an NP/SW team; creation of a comprehensive care plan in consultation with an interdisciplinary care team; and activation of standardized care protocols to address common issues. Care Support team members felt that the patients who appeared to benefit the most were those with complex medical needs, little community support, and mild levels of anxiety, which are known drivers of high health care utilization.[3, 12] Those who benefitted the least were those with more severe mental health issues and lack of interest in engaging with the health care system.

In patients who enrolled in Care Support, health care utilization declined significantly for both ED visits and hospitalizations when comparing utilization 6 months before versus 6 months after enrollment. In addition, patients reported significantly better self-rated health over time after enrolling in Care Support. The impact of Care Support implementation was similar to the original efficacy study, which found decreased acute care utilization and improved self-rated health in those who participated in the program compared to a usual care control group.[6]

There is growing evidence that home-based and team-based care models can improve quality of care while reducing utilization and costs. Although one recent systematic review of preventive home visits from health or social care professionals concluded that they had no effect on mortality, institutionalization or hospitalization and only small effects on function and quality of life,[13] several other systematic reviews and meta-analyses have identified specific aspects of home-based care that are associated with better outcomes. Specifically, beneficial effects of home visits are greater in interventions that include more visits,[14] younger patients[14, 15] and multidimensional assessment.[14, 15] In addition, home-based primary care is most effective when it involves interprofessional care teams that meet regularly and provide after-hours support,[16] all of which are aspects of Care Support.

Similarly, a comparative effectiveness review found that outpatient case management for adults with complex care needs is associated with small improvements in quality of life, quality of care and health care utilization.[17] Characteristics of successful interventions included greater contact time, longer duration, face-to-face visits, and integration with patients’ usual care providers.[17]

A comprehensive synthesis of care management in patients with complex health care needs[18] found that there is “convincing evidence” that care management in primary care improves quality of care, with significant improvements observed in 7 of 9 studies.[6, 19–26] However, only 3 of these studies found significant reductions in utilization and costs, one of which was GRACE.[6, 20, 22] All three of these studies specifically targeted patients with multiple chronic conditions and an increased risk of incurring major health care costs. In addition, they all emphasized training of the care management team, reasonable patient panel sizes, building relationships with PCPs and frequent contacts with patients.[18]

Identifying key elements of care for patients who have complex care needs is becoming particularly relevant as payers and healthcare systems focus on value-based care.^27^ Whereas much of the emphasis of integrated care delivery networks has been on identifying those with the highest need using various types of “analytics,”^28^ an equal amount of attention will need to be directed toward which care elements offer the most benefit to those with a complex array of health and social concerns. The adaptations of GRACE described in this study may be relevant in a number of care settings and, with standardization, could be disseminated widely.

Strengths of our study include the comprehensive evaluation using the CFIR model. Weaknesses include lack of randomization to intervention and control groups, which we attempted to address by using patients as their own controls and comparing utilization during the 6 months before and after enrollment in Care Support. We did not use the 67 patients on the waitlist as controls because implementation of Care Support was associated with changes in the targeted clinics (e.g., staffing, education) that could potentially have had indirect benefits in those not formally enrolled. In addition, our analyses of utilization outside our medical center were based on self-report. Our interdisciplinary team included a mental health professional (PhD psychologist), which may not be available in all healthcare settings; however, this team member primarily served as a consultant to the team, and there is growing awareness of the importance of incorporating mental health to maximize patient well-being. Finally, although utilization decreased, we were unable to perform a formal cost-benefit analysis. The key costs were related to staffing, which included a full-time social worker and full-time nurse practitioner. During implementation, this was increased from one to two teams. The savings due to decreased utilization would be extremely difficult to estimate because different patients had different types of insurance with different payment policies.

In summary, our study suggests that it is feasible to implement the GRACE/Care Support model at an academic medical center by making adaptations based on local needs, and that patients who participated in Care Support experienced significant reductions in acute care utilization and improvements in self-rated health.

## Supporting Information

S1 Appendix Limited dataset. To minimize the risk of loss of privacy for older patients, age has been truncated to a maximum value of 89 years.
(XLS)
